# Dynamic changes in quadriceps and diaphragm thickness for early diagnosis of ICU-acquired weakness in elderly ICU patients: a prospective bedside ultrasound study

**DOI:** 10.3389/fmed.2026.1892704

**Published:** 2026-09-16

**Authors:** Xuehua Chen, Hong Zhou, Tongjuan Zou, Qianrong Ding, Ling Lei, Xia Zhou, Hongxia Li, Wanhong Yin, Yao Chen

**Affiliations:** 1 Department of Critical Care Medicine, Zigong Fourth People's Hospital, Zigong, Sichuan, China; 2 Department of Critical Care Medicine, Affiliated Hospital of Zunyi Medical University, Zunyi, Guizhou,,China; 3 Department of Critical Care Medicine, West China Hospital, Chengdu, China

**Keywords:** bedside ultrasound, diaphragm, elderly, intensive care unit-acquired weakness, quadriceps muscle

## Abstract

**Objective:**

To investigate the value of bedside ultrasound in dynamically monitoring quadriceps and diaphragm thickness for early diagnosis of ICU-acquired weakness (ICU-AW) in elderly critically ill patients.

**Methods:**

This prospective observational study enrolled elderly patients (aged ≥60 years) with an expected ICU stay of ≥7 days. Bedside ultrasound was performed on days 1 (D1), 3, 5, and 7 after ICU admission to measure rectus femoris cross-sectional area (RF-CSA), rectus femoris muscle layer thickness (RF-MLT), vastus intermedius muscle layer thickness (VI-MLT), end-inspiratory diaphragm thickness (DTei), and end-expiratory diaphragm thickness (DTee). Muscle strength was assessed using the Medical Research Council (MRC) sum score. The rates of change of each parameter were compared between the ICU-AW group (MRC ≤ 48) and non-ICU-AW group. Receiver operating characteristic (ROC) curves were used to evaluate predictive performance.

**Results:**

Of 74 enrolled patients, 52 (70.3%) developed ICU-AW. No significant differences in baseline ultrasound parameters were found between groups. The rate of change of RF-MLT on D5 and D7 and the rate of change of DTee on D7 were significantly different between groups (*P* = 0.049, 0.021, and 0.009, respectively). The rate of change of DTee on D7 showed the strongest negative correlation with MRC score (*r* = −0.413, *P* < 0.001). ROC analysis showed that the D7 relative change in DTee had the numerically highest AUC (0.693, 95% CI: 0.556–0.830), with an optimal cutoff value of a decrease ≥6.07% (sensitivity 73.1%, specificity 59.1%), while RF-MLT yielded an AUC of 0.672 with a cutoff value of a decrease ≥3.45% (sensitivity 50.0%, specificity 86.4%). However, the difference between the two AUCs was small (0.021) and not formally tested, and the low specificity (59.1%) limits its clinical utility.

**Conclusion:**

Progressive quadriceps and diaphragm atrophy occurs early in elderly ICU patients. The D7 relative change in DTee showed the numerically highest AUC among the evaluated parameters, suggesting modest discriminatory ability for ICU-AW and warranting further validation. Dynamic muscle ultrasound monitoring is feasible and reproducible at the bedside.

## 1 Introduction

 Intensive care unit-acquired weakness (ICU-AW) is a common neuromuscular complication in critically ill patients, characterized by symmetric limb weakness that cannot be fully explained by the primary illness (1). The incidence of ICU-AW is high among critically ill patients, particularly in those receiving mechanical ventilation. It significantly prolongs the duration of mechanical ventilation, weaning time, length of ICU stay, and total hospital stay, and increases the risk of long-term dysfunction and mortality, severely affecting prognosis and quality of life (2–4). However, patients, aged ≥60 years are particularly vulnerable to muscle wasting and functional decline during critical illness, and due to age-related sarcopenia and reduced physiological reserve, they are at higher risk for developing ICU-AW (5, 6). Therefore, in-depth investigation in this specific population is urgently needed. Early identification of ICU-AW and timely implementation of targeted interventions are of great clinical significance for improving outcomes in elderly critically ill patients (7).

Mechanical ventilation, prolonged immobilization, sedation and analgesia, inflammatory responses, and hypercatabolism are important contributors to the development of ICU-AW (5, 8, 9). Critically ill patients often remain immobilized during their hospital stay, with markedly reduced muscle activity, which rapidly leads to decreased protein synthesis and increased protein breakdown, subsequently resulting in skeletal muscle atrophy and loss of muscle strength (10, 11). Studies have shown that mechanically ventilated patients are at high risk for ICU-AW, and its incidence is closely related to mechanical ventilation (12, 13). The incidence of ICU-AW also varies across different disease states, with higher risk observed in patients with acute respiratory distress syndrome, multiple organ dysfunction, systemic inflammatory response syndrome, septic shock, and coma (13, 14). A recent systematic review indicated that the average prevalence of ICU-AW in critically ill patients is considerably high, highlighting it as a significant issue that cannot be overlooked in ICU management (15, 16).

The established reference standards for diagnosing ICU-AW include the MRC sum score, muscle biopsy, and electrophysiological testing. Emerging tools such as manual muscle testing, imaging, serum biomarkers, and neuromuscular ultrasound have also been explored as alternatives. However, their diagnostic accuracy varies across studies, and each method has its own strengths and limitations (17). Despite the availability of multiple options, the MRC sum score remains the most commonly used clinical diagnostic method due to its practicality and wide acceptance (18–20). Nevertheless, this approach requires patients to be awake, cooperative, and capable of performing active movements, making it susceptible to confounding factors such as impaired consciousness, sedative use, and delirium (21, 22). These inherent limitations severely restrict its applicability in the early stages of critical illness, when patients are often unable to cooperate. Therefore, there is a pressing need for a noninvasive, objective, and reproducible assessment tool that can be reliably performed at the bedside, independent of patient cooperation, to facilitate early identification of ICU-AW (23).

Muscle ultrasound, as a noninvasive, real-time, and reproducible bedside technique, has gained increasing attention for assessing muscle status in critically ill patients in recent years (19, 24). The quadriceps muscle is a peripheral muscle group that is relatively sensitive to systemic skeletal muscle loss, and changes in its thickness can indicate early muscle wasting (25). Meanwhile, the diaphragm, as the primary respiratory muscle, exhibits structural changes that are closely associated with respiratory muscle weakness and weaning failure, and may also participate in the development and progression of ICU-AW (26–28). Compared with assessing peripheral skeletal muscle alone, simultaneous monitoring of dynamic changes in both the quadriceps and diaphragm may more comprehensively reflect the injury process of both peripheral and respiratory muscles in critically ill patients, thereby providing a more reliable basis for early identification of ICU-AW (29, 30). Despite its limitations, the MRC sum score remains the most recognized clinical reference standard for diagnosing ICU-AW. This study used bedside ultrasound to dynamically measure changes in quadriceps and diaphragm thickness in elderly critically ill patients, explored their correlation with muscle strength, and evaluated their value in the early diagnosis of ICU-AW.

## 2 Methods and participants

### 2.1 Study design

This was a prospective observational study conducted at the Department of Critical Care Medicine, Zigong Fourth People's Hospital, Zigong, China, from December 2023 to January 2025, and was reported in accordance with the Strengthening the Reporting of Observational Studies in Epidemiology (STROBE) statement.

### 2.2 Study participants

A convenience sampling method was used to recruit participants. After ICU admission, the research team explained the study protocol to eligible patients and their family members and assessed their willingness to participate. The inclusion and exclusion criteria were strictly reviewed, and reasons for exclusion were documented. Eligible patients who provided signed written informed consent were enrolled. Ethical guidelines were followed throughout to protect patient rights, and complete records were maintained. The specific inclusion and exclusion criteria were as follows:

#### 2.2.1 Inclusion criteria

1. Patients had a Barthel Index (BI) score of ≥60 for activities of daily living prior to ICU admission.
2. Patients were expected to stay in the ICU for ≥7 days and were aged ≥60 years.
3. Patients had no organic brain lesions, lower limb motor dysfunction, or neurological disorders affecting motor function, such as neurodegenerative diseases, spinal cord lesions, peripheral neuropathies [e.g., severe diabetic peripheral neuropathy and Guillain-Barré syndrome (GBS)], myasthenia gravis, multiple sclerosis, Parkinson's disease, or other conditions affecting motor function; and no trauma or surgery involving the lumbar spine, pelvis, or lower limbs, or limb defects.
4. Patients voluntarily participated after communication with their family members.

#### 2.2.2 Exclusion criteria

1. Presence of skin damage, redness, swelling, bleeding, or other conditions affecting ultrasound imaging in the lower limbs or diaphragm areas.
2. Expected death, receiving palliative care, or incomplete data collection.

### 2.3 Sample size and statistical power

The sample size was estimated using the Hanley & McNeil method (31), with the area under the curve (AUC) as the primary outcome measure. The expected AUC was set at 0.75, with a two-sided type I error (*α*) of 0.05 and a type II error (*β*) of 0.20 (powe*r* = 0.80). Based on previous literature, the reported incidence of ICU-AW ranges from 25% to 100% (32, 33); in this study, it was assumed to be 70%. The required total sample size was calculated using the following formula:

$$N=\frac{{\left(Z_{\alpha /2}+Z_{\beta }\right)}^{2}\;\times \frac{\mathrm{Var}(\mathrm{AUC})}{\;p(1-p)}}{{(\mathrm{AUC}-0.5)}^{2}}\;\times \frac{1}{1\text{-}\mathrm{dropout}\;\mathrm{rate}}$$

where Z*α*/2 = 1.96, Z*β* = 0.84, *p* = 0.70, Var(AUC) = 0.079, and dropout rate = 0.20. The calculated minimum required sample size was 68 patients.

In the present study, 74 patients were ultimately included in the final analysis, including 52 patients with ICU-AW and 22 without ICU-AW. A *post hoc* power analysis was performed based on the observed AUC of the best-performing diagnostic indicator, namely the rate of change of end-expiratory diaphragm thickness (DTee) on Day 7. With an observed AUC of 0.693, a two-sided *α* level of 0.05, and the final sample size of 74, the achieved statistical power was 86.1%, confirming that the final sample size had adequate power to detect the observed diagnostic effect.

### 2.4 Data collection and outcome measures

#### 2.4.1 Baseline data

At enrollment, the following data were collected: age, sex, body mass index (BMI), primary diagnosis, Acute Physiology and Chronic Health Evaluation II (APACHE II) score, Nutrition Risk in Critically Ill (NUTRIC) score (modified NUTRIC score, mNUTRIC), Sequential Organ Failure Assessment (SOFA) score, nutritional support method, ventilation mode, early mobilization status.

#### 2.4.2 Ultrasound measurements

Using a Mindray ultrasound device (Mindray, Shenzhen, China) equipped with a 5–10 MHz linear array probe for bedside B-mode ultrasonography, with the following settings: imaging depth standardized with the femur positioned at the center-third of the screen as the reference, gain set to 50%, focal zone placed at the target muscle level, and transducer frequency of 5–10 MHz. These settings were maintained consistently for each patient across all measurement time points (Days 1, 3, 5, and 7) using the same ultrasound device and fixed parameters. Ultrasound measurements were performed by two trained intensive care nurses [with Chinese Critical Care Ultrasound Study Group (CCUSG) certification]. Measurements were taken at the specific time points (Day 1, Day 3, Day 5, Day 7) after ICU admission. Each parameter was measured three times, and the average value was used for analysis. Patients were placed in the supine position; if there was a risk of gastroesophageal reflux, the head of the bed was elevated to 30–45°. Both lower limbs were extended and relaxed, with toes pointing toward the ceiling. Care was taken to maintain midline body alignment, avoiding lateral rotation or pelvic tilt. External rotation and abduction of the lower limbs, which could affect Rectus Femoris (RF) imaging, were avoided.

The sampling point for quadriceps measurement was located at the middle-lower third of the line between the anterior superior iliac spine and the superior border of the patella. A skin marker was used to mark the sampling point or its horizontal line to ensure consistent measurement site and time. Both quadriceps were measured; as no difference was observed between the left and right sides, data from the right side were selected for reporting. The following parameters were measured without compression: Right Rectus Femoris Cross-Sectional Area (RF-CSA, cm^2^), RF-MLT (cm), and Vastus Intermedius Muscle Layer Thickness (VI-MLT, cm), This measurement protocol is consistent with a previously published multicentre study (34).

For Diaphragm Thickness at End-Inspiration/Expiration (DTei/DTee) measurement, patients remained in the supine position. Measurements were performed at end-expiration and end-inspiration using M-mode ultrasound with a frame rate of 10 mm/s to visualize diaphragmatic motion and identify respiratory phases. End-inspiratory diaphragm thickness was measured at the widest point between the two pleural-peritoneal lines, while end-expiratory thickness was measured at the narrowest point within the same respiratory cycle, with measurement markers placed close to the inner diaphragmatic membranes. Ventilator settings (mode, PEEP, pressure support, and tidal volume) were not recorded in this study, as these parameters were determined by clinical requirements. The sampling point was the right diaphragm zone of apposition (ZOA), typically at the 8th or 9th intercostal space near the anterior axillary line. The same skin marker was used to ensure reproducibility. The following parameters were measured without compression: DTei (cm) and DTee (cm). Diaphragmatic excursion was not measured, as we focused on structural thickness changes, which are more feasible and reproducible in this population. A complete ultrasound examination, including all quadriceps and diaphragm parameters, required approximately 10–15 min per patient per time point. The specific measurement methods and timing are shown in Figure 1.

**Figure 1:**
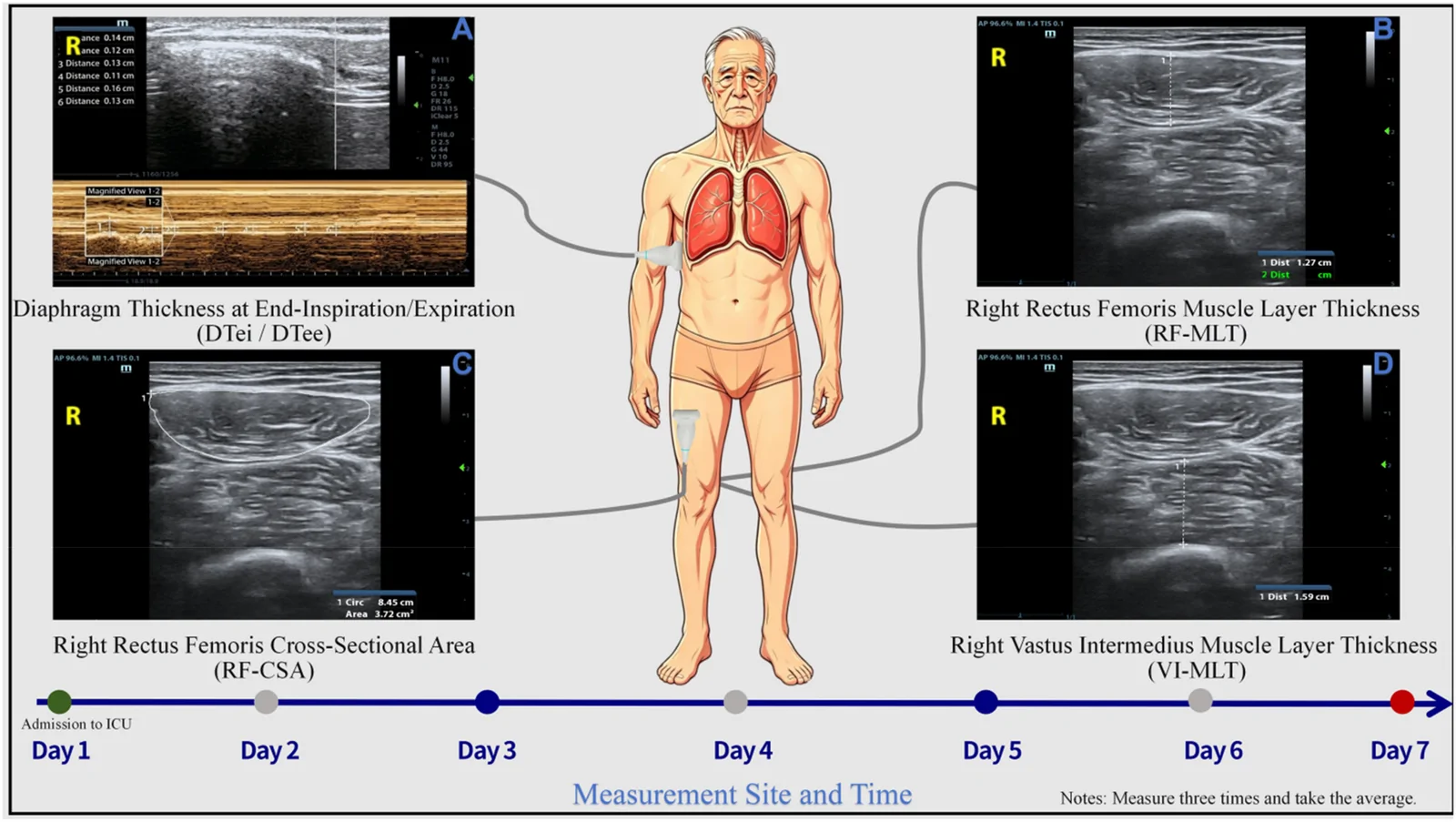
Measurement methods and timing of quadriceps and diaphragm ultrasound parameters.

#### 2.4.3 Muscle strength assessment

Muscle strength was assessed using the Medical Research Council (MRC) sum score, which includes 12 items (e.g., elbow extension, wrist extension, knee flexion), with a total score ranging from 0 (quadriplegia) to 60 (normal muscle strength). The assessment was performed by a nurse with at least five years of ICU nursing experience at the time of patient enrollment and discharge. The assessment was conducted only when patients had a Richmond Agitation-Sedation Scale (RASS) score between −1 and 1, were judged by the assessing nurse to be able to consistently follow commands, and were non-delirious. ICU-AW was diagnosed when the MRC score was ≤48.

#### 2.4.4 Outcome measures

The primary outcome measure was the diagnostic efficacy of dynamic changes in quadriceps and diaphragm ultrasound parameters for ICU-AW. An MRC sum score ≤48 was used as the diagnostic reference standard. Receiver operating characteristic (ROC) curve analysis was performed to evaluate the diagnostic value of the rate of change of each muscle parameter for ICU-AW. The AUC, optimal cutoff value, sensitivity, and specificity were calculated.

The secondary outcome measures included: (1) dynamic changes in RF-CSA, RF-MLT, VI-MLT, DTei, and DTee on days 1, 3, 5, and 7 of ICU stay; (2) correlations between the rate of change of each muscle parameter and the MRC score; and (3) comparisons of the rate of change of each muscle parameter between the ICU-AW group and the non-ICU-AW group.

### 2.5 Quality control

The same ultrasound device was used throughout the study. All ultrasound measurements were performed by two trained intensive care nurses who held CCUSG certification and had extensive work experience, following a standardized protocol. This study is a sub-center project of the multi-center study titled “Visualized Evaluation System for Critical Illness-Acquired Weakness.” A pre-test conducted on 10 patients before formal implementation showed good inter-observer agreement [intraclass correlation coefficient (ICC) > 0.85], and intra-observer reliability assessed by the same operator repeating measurements at 24-h intervals in 10 cases also showed good agreement (ICC > 0.90). All operators received standardized training on data entry procedures and entered the data into the multi-center database system after logging in with assigned credentials. At each time point (days 1, 3, 5, and 7 after ICU admission), each parameter was measured three times, and the average value was used for analysis. The same skin marker was used to ensure consistent probe placement. MRC scores were assessed by an experienced ICU nurse who was blinded to the ultrasound results, and assessments were performed when patients were cooperative and non-delirious (RASS score between −1 and 1). All data were entered by two individuals independently and cross-checked.

### 2.6 Statistical analysis

Statistical analysis was performed using SPSS version 29.0. Continuous data were expressed as mean ± standard deviation or median (interquartile range, IQR) when distribution was skewed, and categorical data as frequencies and percentages. Group comparisons were performed using the *t*-test or Mann–Whitney *U*-test. Changes in muscle parameters over different time points were analyzed using one-way repeated-measures analysis of variance. Before conducting repeated-measures ANOVA, the assumptions of normality and sphericity were assessed. Sphericity was evaluated using Mauchly's test. When the assumption of sphericity was violated, Greenhouse–Geisser correction was applied to adjust the degrees of freedom. Pairwise comparisons between time points were performed using the Bonferroni correction. Partial eta squared (*η*p^2^) was calculated as the effect size for repeated-measures ANOVA. The strength of correlations was interpreted as follows based on established guidelines: |*r*| < 0.3 indicated a weak correlation, 0.3 ≤ |*r*| < 0.5 indicated a moderate correlation, and |*r*| ≥ 0.5 indicated a strong correlation. A *P*-value < 0.05 was considered statistically significant.

## 3 Results

### 3.1 General information of participants

A total of 940 ICU patients were initially screened. After screening according to the inclusion and exclusion criteria, 74 elderly patients were finally included in the analysis. The participant enrollment and screening process is shown in Figure 2.

**Figure 2:**
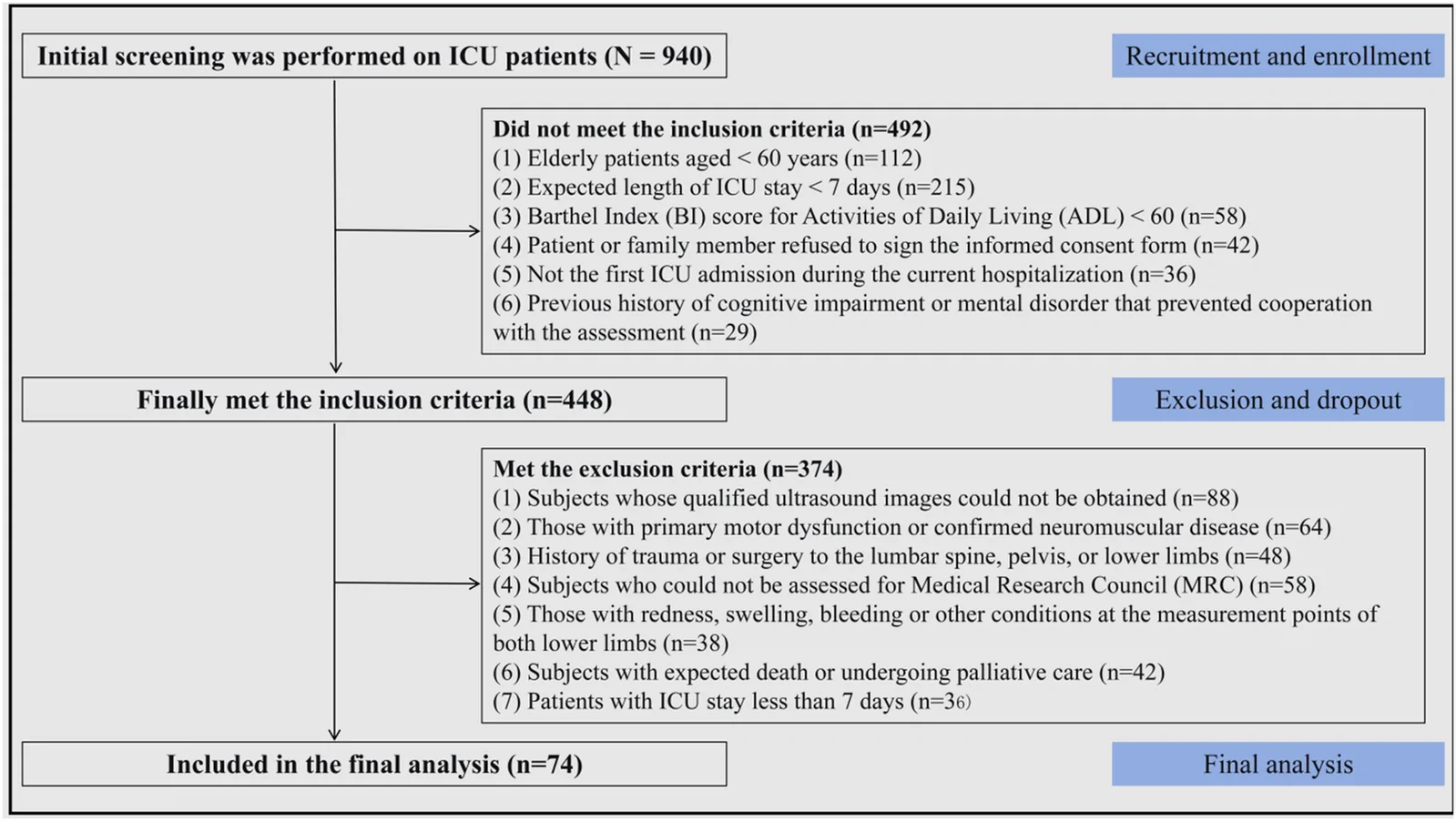
Participant enrollment and screening flow chart.

In 74 elderly intensive care unit (ICU) patients (66.2% male), mean age was 76.55 ± 7.79 years, APACHE II score 23.45 ± 5.90, mNUTRIC score 6.23 ± 1.56, BMI 22.52 ± 3.26 kg/m^2^, and ICU stay 8.30 ± 1.60 days. Primary diagnoses: respiratory 37 (50.0%), nervous system 11 (14.9%), trauma/orthopedic 7 (9.5%), digestive 5 (6.8%), other 14 (18.9%). For nutritional support, combined parenteral nutrition (PN) and enteral nutrition (EN) was used in 68 (81.1%). Oxygen therapy: invasive mechanical ventilation in 68 (91.9%). Early mobilization: passive bedside in 45 (60.8%), active bedside/sitting in 29 (39.2%). Detailed information is presented in Table 1.

**Table 1:** General information of enrolled elderly ICU patients (*N* = 74).

Item	*n* (%)	$\bar{X}\;\pm s$
Gender		
Male	49 (66.2)	
Female	25 (33.8)	
Age (years)		76.55 ± 7.79
APACHE II score		23.45 ± 5.90
mNUTRIC score		6.23 ± 1.56
BMI (kg/m^2^)		22.52 ± 3.26
ICU length of stay (days)		8.30 ± 1.60
Primary diagnosis		
Respiratory system disease	37 (50.0)	
Nervous system disease	11 (14.9)	
Trauma/orthopedic disease	7 (9.5)	
Digestive system disease	5 (6.8)	
Other	14 (18.9)	
Nutritional support mode		
PN (parenteral nutrition)	14 (18.9)	
PN + EN (enteral nutrition)	68 (81.1)	
Oxygen therapy mode		
Invasive mechanical ventilation	68 (91.9)	
Face mask oxygen	3 (4.1)	
Nasal cannula oxygen	2 (2.7)	
High-flow nasal cannula oxygen therapy	1 (1.3)	
Early mobilization		
Passive bedside exercise	45 (60.8)	
Active bedside/sitting exercise	29 (39.2)	

APACHE II, Acute Physiology and Chronic Health Evaluation II; mNUTRIC, Nutrition Risk in the Critically Ill; BMI, body mass index.

### 3.2 Changes in quadriceps and diaphragm thickness during ICU stay

From Day 1 (D1) to Day 7 (D7) after ICU admission, the quadriceps and diaphragm ultrasound parameters showed an overall decreasing tendency. Although slight fluctuations were observed in RF-MLT after D5 and in DTei after D3, the overall D1-to-D7 changes indicated a net decrease in muscle and diaphragm thickness. Repeated-measures ANOVA showed significant overall time effects for RF-CSA, RF-MLT, VI-MLT, DTei, and DTee (F = 60.751, 26.864, 33.983, 50.619, and 81.693, respectively; all corrected *P* < 0.001), as shown in Table 2. The mean change trends are presented in Figure 3.

**Table 2:** Changes in quadriceps and diaphragm thickness in elderly patients during ICU stay.

Parameter	Day 1	Day 3	Day 5	Day 7	*η*p^2^	*P* value
RF-CSA (cm^2^)	2.30 ± 0.83^a^	2.25 ± 0.78^b^	2.21 ± 0.76^c^	2.19 ± 0.75^d^	0.194	<0.001
RF-MLT (cm)	0.63 ± 0.25^a^	0.61 ± 0.24^b^	0.60 ± 0.24^c^	0.61 ± 0.25^d^	0.177	<0.001
VI-MLT (cm)	0.69 ± 0.30^a^	0.67 ± 0.28^a^	0.66 ± 0.27^b^	0.65 ± 0.27^c^	0.155	<0.001
DTei (cm)	0.19 ± 0.04^a^	0.19 ± 0.04^b^	0.18 ± 0.04^c^	0.18 ± 0.04^d^	0.448	<0.001
DTee (cm)	0.18 ± 0.04^a^	0.18 ± 0.04^b^	0.17 ± 0.04^c^	0.17 ± 0.04^d^	0.485	<0.001

Data are presented as mean ± standard deviation. *P* values refer to the overall time effect assessed by repeated-measures ANOVA. Partial eta squared (ηp^2^) was used as the effect size measure. Different superscript letters within the same row indicate statistically significant differences between time points based on Bonferroni-adjusted pairwise comparisons (*P* < 0.05), whereas the same superscript letter indicates no statistically significant difference. RF-CSA, rectus femoris cross-sectional area; RF-MLT, rectus femoris muscle layer thickness; VI-MLT, vastus intermedius muscle layer thickness; DTei, diaphragm thickness at end-inspiration; DTee, diaphragm thickness at end-expiration.

**Figure 3:**
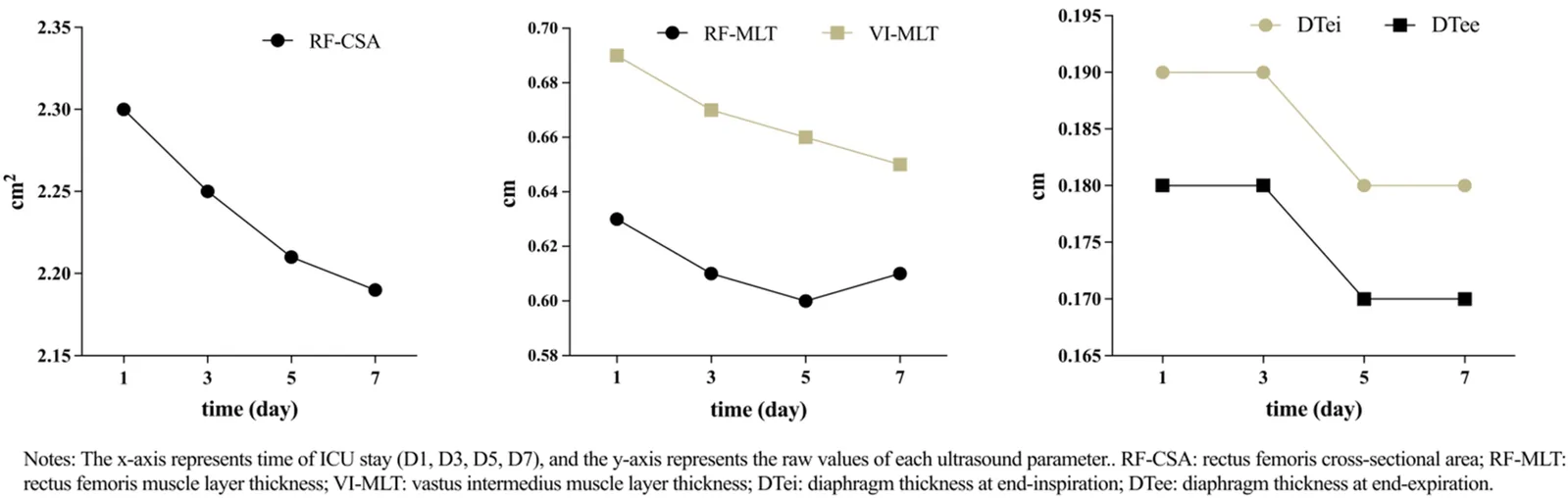
Dynamic changes in quadriceps and diaphragm thickness over time.

### 3.3 Dynamic changes in quadriceps

As shown in Table 3, compared with D1, the quadriceps ultrasound parameters showed an overall decreasing tendency at D3, D5, and D7. The absolute changes showed that by D7, RF-CSA, RF-MLT, and VI-MLT decreased by −0.11 ± 0.21 cm^2^, −0.03 ± 0.06 cm, and −0.04 ± 0.08 cm, respectively. The decreases in RF-CSA, RF-MLT, and VI-MLT from D1 to D3, D5, and D7 were statistically significant after adjustment for multiple comparisons, as shown in Table 3. The relative change rate analysis showed that by D7, RF-CSA, RF-MLT, and VI-MLT decreased by 3.92%, 3.85%, and 3.95%, respectively.

**Table 3:** Dynamic changes of quadriceps in elderly patients during ICU hospitalization.

TIME	RF-CSA (cm^2^)	RF-MLT (cm)	VI-MLT (cm)
D3–D1	−0.05 ± 0.13	−0.02 ± 0.06	−0.02 ± 0.07
(D3–D1)/D1 (%)	−2.00 (−2.51, −1.30)	−1.98 (−2.57, −1.25)	−1.96 (−2.64,−1.34)
D5–D1	−0.09 ± 0.20	−0.03 ± 0.06	−0.03 ± 0.08
(D5–D1)/D1 (%)	−3.03 (−3.53, −2.32)	−3.23 (−4.00, −2.46)	−3.20 (−3.69, −2.63)
D7–D1	−0.11 ± 0.21	−0.03 ± 0.06	−0.04 ± 0.08
(D7–D1)/D1 (%)	−3.92 (−4.55, −3.24)	−3.85 (−4.72, −3.21)	−3.95 (−5.00, −3.21)

RF-CSA, rectus femoris cross-sectional area; RF-MLT, rectus femoris muscle layer thickness; VI-MLT, vastus intermedius muscle layer thickness; D1, D3, D5, D7 represent the values on days 1, 3, 5, and 7 of ICU admission, respectively; Relative change values are expressed as percentages (%) and were calculated as [(D*x*−D1)/D1] × 100%, where *x* represents D3, D5, or D7. Negative values indicate a decrease from baseline. The overall differences in the rate of change across different time points for each parameter were statistically significant (all *P* < 0.001).

### 3.4 Dynamic changes in diaphragm

As shown in Table 4, diaphragm thickness also showed a gradual decreasing trend with ICU stay. The absolute changes showed that by D7, both DTei and DTee decreased by approximately 0.01 cm. The relative change rates showed that by D7, DTei and DTee decreased by approximately 5% and 6%, respectively, indicating a clear atrophic trend in the diaphragm, with a slightly greater decrease in end-expiratory diaphragm thickness.

**Table 4:** Dynamic changes in diaphragm in elderly patients during ICU stay.

TIME	DTei (cm)	DTee (cm)
D3–D1	−0.00 ± 0.01	−0.00 ± 0.01
(D3–D1)/D1 (%)	−0.00 (−0.04, −0.00)	−0.00 (−0.00, −0.00)
D5–D1	−0.01 ± 0.01	−0.01 ± 0.01
(D5–D1)/D1 (%)	−0.05 (−0.06, −0.00)	−0.04 (−0.06, −0.00)
D7–D1	−0.01 ± 0.01	−0.01 ± 0.01
(D7–D1)/D1 (%)	−0.05 (−0.06, −0.04)	−0.06 (−0.06, −0.04)

DTei, diaphragm thickness at end-inspiration; DTee, diaphragm thickness at end-expiration; D1, D3, D5, D7 represent the values on days 1, 3, 5, and 7 of ICU admission, respectively; Relative change values are expressed as percentages (%) and were calculated as [(Dx−D1)/D1] × 100%, where x represents D3, D5, or D7. Negative values indicate a decrease from baseline. Adjusted *P* values refer to comparisons with D1. All changes from D1 to D3, D5, and D7 were statistically significant after adjustment for multiple comparisons (all adjusted *P* < 0.001).

Figure 4 shows the rate of change of quadriceps and diaphragm ultrasound parameters at different time points relative to baseline (D1) in ICU patients. The results showed that RF-CSA, RF-MLT, VI-MLT, DTei, and DTee all showed a decreasing trend at D3 and D7 compared with D1, with the overall rate of change below 0. Compared with D3, the rate of change of the above parameters further decreased at D7, and the differences were statistically significant (all *P* < 0.001), suggesting that the degree of quadriceps and diaphragm atrophy gradually worsened with prolonged ICU stay.

**Figure 4:**
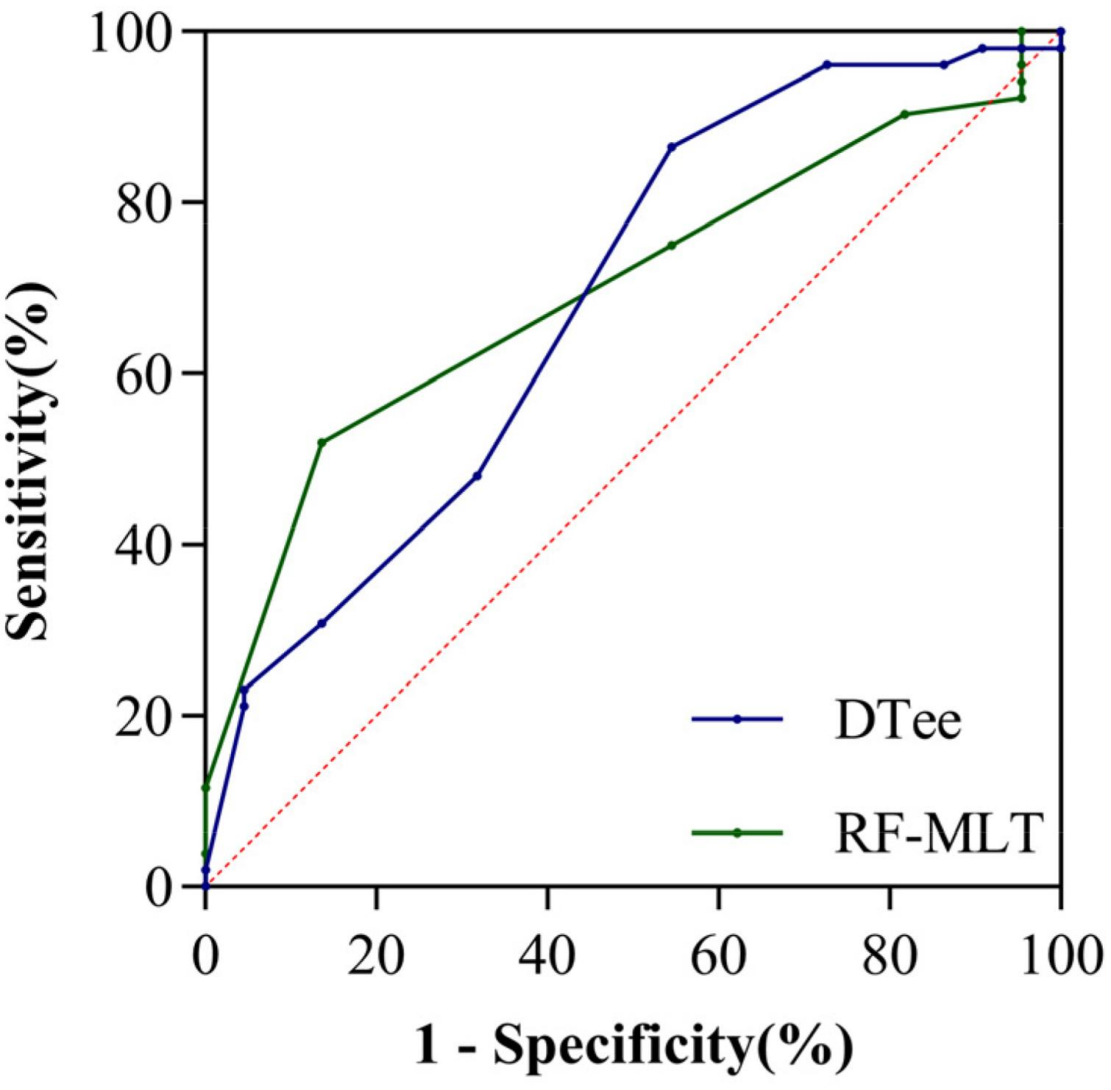
ROC curves of D7 relative changes in RF-MLT and DTee for discriminating ICU-AW. The blue curve represents DTee and the green curve represents RF-MLT. The red dashed diagonal line indicates an AUC of 0.5 (no discrimination).

### 3.5 Comparison between ICU-AW and Non-ICU-AW patients

As shown in Table 5, a total of 74 critically ill patients were enrolled in this study, of whom 52 (70.3%) developed ICU-AW and 22 (29.7%) did not. Comparison of baseline muscle parameters showed that on Day 1 (D1) of ICU admission, there were no statistically significant differences in RF-CSA, RF-MLT, VI-MLT, DTei, or DTee between the two groups (all *P* > 0.05), indicating good comparability of muscle ultrasound parameters upon ICU admission.

**Table 5:** Comparison of muscle ultrasound parameters between ICU-AW and Non-ICU-AW elderly patients.

Parameter	ICU-AW (*n* = 52)	Non-ICU-AW (*n* = 22)	*t/Z* value	*P* value
MRC score	42.54 ± 8.57	54.68 ± 3.80	−6.37^a^	<.001
D1RF-CSA (cm^2^)	2.24 (1.77, 2.85)	2.03 (1.51, 2.70)	−0.822^b^	0.411
D1RF-MLT (cm)	0.62 (0.43, 0.80)	0.64 (0.38, 0.80)	−0.242^b^	0.808
D1VI-MLT (cm)	0.62 (0.43, 0.80)	0.69 (0.55, 0.86)	−0.946^b^	0.344
D1DTei	0.18 ± 0.03	0.21 ± 0.05	1.604^a^	0.209
D1DTee	0.18 ± 0.04	0.18 ± 0.04	0.375^a^	0.542
RF-CSA (%)				
(D3–D1)/D1	−0.02 (−0.03, −0.01)	−0.02 (−0.03, −0.02)	−0.254^b^	0.799
(D5–D1)/D1	−0.03 (−0.04, −0.02)	−0.03 (−0.04, −0.03)	−1.053^b^	0.293
(D7–D1)/D1	−0.04 (−0.04, −0.03)	−0.04 (−0.05, −0.04)	−1.632^b^	0.103
RF-MLT (%)				
(D3–D1)/D1	−0.02 (−0.02, −0.01)	−0.02 (−0.03, −0.01)	−0.729^b^	0.466
(D5–D1)/D1	−0.03 (−0.04, −0.02)	−0.03 (−0.04, −0.03)	−1.969^b^	**0** **.** **049**
(D7–D1)/D1	−0.03 (−0.05, −0.03)	−0.04 (−0.05, −0.04)	−2.301^b^	**0** **.** **021**
VI-MLT (%)				
(D3–D1)/D1	−0.02 (−0.03, −0.01)	−0.02 (−0.03, −0.01)	−0.207^b^	0.836
(D5–D1)/D1	−0.03 (−0.04, −0.03)	−0.04 (−0.04, −0.03)	−1.916^b^	0.055
(D7–D1)/D1	−0.04 (−0.05, −0.03)	−0.04 (−0.05, −0.04)	−1.780^b^	0.075
DTei (%)				
(D3–D1)/D1	0.00 (0.00, 0.00)	0.00 (−0.04, 0.00)	−1.179^b^	0.239
(D5–D1)/D1	−0.04 (−0.06, 0.00)	−0.05 (−0.05, −0.04)	−0.163^b^	0.871
(D7–D1)/D1	−0.05 (−0.06, −0.04)	−0.05 (−0.06, −0.04)	−0.279^b^	0.780
DTee (%)				
(D3–D1)/D1	0.00 (0.00, 0.00)	0.00 (−0.01, 0.00)	−1.021^b^	0.307
(D5–D1)/D1	−0.01 (−0.06, 0.00)	−0.04 (−0.06, 0.00)	−1.027^b^	0.304
(D7–D1)/D1	−0.06 (−0.06, −0.04)	−0.06 (−0.08, −0.05)	−2.626^b^	**0** **.** **009**

RF-CSA, rectus femoris cross-sectional area; RF-MLT, rectus femoris muscle layer thickness; VI-MLT, vastus intermedius muscle layer thickness; DTei, diaphragm thickness at end-inspiration; DTee, diaphragm thickness at end-expiration; D1, D3, D5, D7 represent the values on days 1, 3, 5, and 7 of ICU admission, respectively;*P* values < 0.05 are shown in bold.

a independent-sample *t*-test.

b Mann–Whitney *U*-test.

Further comparison of the rate of change relative to baseline (Day 1) of each muscle parameter revealed that the rate of change of RF-MLT on D5 and D7 and the rate of change of DTee on D7 were significantly different between the two groups (*P* = 0.049, 0.021, and 0.009, respectively). This suggests that, compared with baseline muscle parameters, dynamic changes in certain muscle parameters may better reflect the occurrence of ICU-AW.

The quadriceps and diaphragm ultrasound parameters in both the ICU-AW and non-ICU-AW groups showed a decreasing trend over time, indicating that critically ill patients experience progressive muscle loss early during ICU hospitalization. The two groups had generally similar muscle parameter levels at D1, but with prolonged ICU stay, the trends of certain indicators gradually diverged, with RF-MLT and DTee showing more significant intergroup differences in the later stage. Compares the rate of change of ultrasound parameters with statistically significant differences between the two groups. The results showed that the rates of change of RF-MLT on D5 and D7 and DTee on D7 were statistically significant between the two groups, suggesting that dynamic changes in rectus femoris thickness and end-expiratory diaphragm thickness may be more closely associated with the occurrence of ICU-AW.

### 3.6 Correlation analysis with muscle strength

As shown in Table 6, correlation analysis revealed that the rate of change relative to baseline (Day 1) of RF-CSA on D5 (*r* = −0.200, *P* = 0.008) and D7 (*r* = −0.255, *P* = 0.028) was negatively correlated with the MRC score, indicating weak correlations. The rate of change of RF-MLT on D5 (*r* = −0.314, *P* = 0.006) and D7 (*r* = −0.332, *P* = 0.004) also showed significantly negative correlations with the MRC score, indicating moderate correlations. The rate of change of VI-MLT on D5 (*r* = −0.247, *P* = 0.034) and D7 (*r* = −0.238, *P* = 0.041) was negatively correlated with the MRC score, indicating weak correlations.

**Table 6:** Correlation analysis between changes in muscle parameters and MRC score.

Variable	*R* value	*P* value
D1 RF-CSA (%)	−0.132	0.263
D5 RF-CSA (%)	−0.200	0.008
D7 RF-CSA (%)	−0.255	0.028
D3 RF-MLT (%)	−0.177	0.130
D5 RF-MLT (%)	−0.314	0.006
D7 RF-MLT (%)	−0.332	0.004
D3 VI-MLT (%)	0.035	0.769
D5 VI-MLT (%)	−0.247	0.034
D7 VI-MLT (%)	−0.238	0.041
D7 DTei (%)	−0.132	0.263
D7 DTee (%)	−0.413	<0.001

RF-CSA, rectus femoris cross-sectional area; RF-MLT, rectus femoris muscle layer thickness; VI-MLT, vastus intermedius muscle layer thickness; DTei, diaphragm thickness at end-inspiration; DTee, diaphragm thickness at end-expiration. All percentage change values are calculated relative to baseline (Day 1). Correlation coefficients (r) were interpreted as follows based on established literature: |*r*| < 0.3 indicated a weak correlation, 0.3 ≤ |*r*| < 0.5 indicated a moderate correlation, and |*r*| ≥ 0.5 indicated a strong correlation.

Regarding the diaphragm, the rate of change of DTee on D7 showed a moderate negative correlation with the MRC score (*r* = −0.413, *P* < 0.001), while no significant correlation was observed for the rate of change of DTei (*r* = −0.132, *P* = 0.263). These findings suggest that a greater degree of muscle and diaphragm atrophy is associated with worse muscle strength, with the strongest correlation observed for changes in end-expiratory diaphragm thickness.

### 3.7 ROC analysis

As shown in Table 7 and Figure 4, ROC analysis was performed to evaluate the discriminatory performance of the D7 relative changes in RF-MLT and DTee for ICU-AW. The *P* values refer to the test of whether the AUC was significantly different from 0.5 (DeLong method); no formal pairwise comparison between AUCs was performed. A parameter was considered to have statistically significant discriminatory ability when the AUC was greater than 0.5 and the 95% CI did not include 0.5. As shown in Table 5, only RF-MLT at D5 and D7 and DTee at D7 showed significant intergroup differences (*P* < 0.05); therefore, the ROC analysis was focused on these two parameters. The D7 relative change in DTee showed a numerically higher AUC than RF-MLT, with an AUC of 0.693 (95% CI: 0.556–0.830). The optimal cutoff value of relative change was ≤−6.07%, with a sensitivity of 73.10% and a specificity of 59.10%. The D7 relative change in RF-MLT yielded an AUC of 0.672 (95% CI: 0.541–0.802). The optimal cutoff value of relative change was ≤−3.45%, with a sensitivity of 50.00% and a specificity of 86.40%. DTee showed the highest numerical AUC (0.693), yet the small difference from RF-MLT (Δ = 0.021) was not formally compared, and its low specificity (59.1%) limits clinical utility.

**Table 7:** ROC analysis of the D7 rate of change in muscle and diaphragm parameters for discriminating ICU-AW.

Parameter	AUC	95%CI	Optimal cutoff value of relative change (%)	Sensitivity (%)	Specificity (%)
RF-MLT (D7-D1)/D1	0.672	0.541–0.802	≤−3.45%	50.00	86.40
DTee (D7-D1)/D1	0.693	0.556–0.830	≤−6.07%	73.10	59.10

AUC, area under the curve; CI, confidence interval; RF-MLT, rectus femoris muscle layer thickness; DTee, diaphragm thickness at end-expiration. Relative change was calculated as [(D7−D1)/D1] × 100%. Therefore, the optimal cutoff values are expressed as percentages (%). Negative values indicate a decrease from baseline. *P* values refer to the test of whether the AUC was significantly different from 0.5 (DeLong method). No formal pairwise comparison between AUCs was performed.

## 4 Discussion

### 4.1 Clinical significance

Bedside ultrasound can dynamically monitor changes in muscles and the diaphragm, offering advantages such as non-invasiveness, good reproducibility, and ease of operation. Compared with the traditional MRC score, it does not depend on the patient's level of consciousness or active cooperation, making it more suitable for early assessment of critically ill patients (34, 35). Combined monitoring of quadriceps and diaphragm changes can comprehensively reflect structural alterations in both peripheral skeletal muscles and the respiratory muscle, providing an objective basis for the early identification of ICU-AW. Previous studies have demonstrated that higher rehabilitation intensity and adequate nutrition are associated with a lower incidence of ICU-AW (36, 37), and that early mobilization combined with high-protein nutrition reduces muscle volume loss (38). Therefore, early identification of high-risk patients through ultrasound monitoring may have important clinical implications for guiding rehabilitation interventions and optimizing nutritional support. In our center, ultrasound measurements were performed by trained rehabilitation therapists (with CCUSG certification) rather than ICU physicians, which makes this approach feasible in routine clinical practice without imposing an excessive workload on busy ICU staff.

### 4.2 Progressive muscle and diaphragm atrophy in ICU patients

Critically ill patients often experience reduced muscle protein synthesis and increased protein breakdown due to multiple factors, including prolonged bed rest and immobilization, mechanical ventilation support, systemic inflammatory response, hypercatabolism, and inadequate nutritional intake, leading to rapid muscle loss and decreased muscle strength (39, 40). Previous studies have shown that the incidence of ICU-AW in critically ill patients is high, ranging from 25% to 100%, and significant skeletal muscle atrophy can occur shortly after ICU admission (41, 42).

The results of this study showed that both quadriceps and diaphragm thickness progressively decreased with prolonged ICU stay, with statistically significant overall time effects confirmed by repeated-measures ANOVA (all *P* < 0.001). *Post-hoc* pairwise comparisons with Bonferroni correction further demonstrated that the decreases from baseline (Day 1) to D3, D5, and D7 were statistically significant for all parameters (all adjusted *P* < 0.001, as shown in Tables 3, 4), suggesting that critically ill patients may develop significant peripheral skeletal muscle and respiratory muscle atrophy early after ICU admission. These findings are generally consistent with previous studies (43). In this study, by D7, quadriceps parameters decreased by approximately 3%–4% from baseline, further supporting the view that progressive muscle wasting occurs early in critically ill patients. This early muscle atrophy pattern is consistent with findings from a multicentre study, which demonstrated that quadriceps muscle thickness and cross-sectional area within 24 h of ICU admission were significantly lower in patients who subsequently developed ICU-AW (34).

Of note, the baseline values of rectus femoris and vastus intermedius thickness in our elderly cohort suggested relatively limited muscle reserve at ICU admission. This finding may be related to age-related sarcopenia and reduced physiological reserve, as well as the relatively smaller body habitus of the southern Chinese population compared with other populations (44). Previous studies in elderly critically ill patients have similarly shown that low skeletal muscle mass at ICU admission is associated with subsequent muscle loss and adverse functional outcomes (45–48). Thus, limited muscle reserve at admission may increase susceptibility to critical illness-related muscle wasting and may partly explain the high incidence of ICU-AW (70.3%) observed in this cohort. However, as pre-illness muscle measurements were not available, this interpretation should be considered a possible explanation rather than a causal inference.

In addition to peripheral skeletal muscle, the diaphragm, as the primary respiratory muscle, is also prone to structural alterations under critical illness conditions. This study found that diaphragm thickness gradually decreased with prolonged ICU stay, with a more pronounced decline in end-expiratory diaphragm thickness (DTee), suggesting that respiratory muscle structural atrophy is also an important manifestation of muscle loss in critically ill patients. Although diaphragmatic function was not directly assessed in this study, structural atrophy of the diaphragm may be associated with clinically relevant outcomes such as weaning failure and prolonged ICU stay. Therefore, dynamic monitoring of diaphragm thickness changes helps to more comprehensively assess the process of muscle wasting in critically ill patients.

### 4.3 Relationship between muscle and diaphragm changes and ICU-AW

Previous studies have shown that prolonged mechanical ventilation and extended ICU stay are important risk factors for ICU-AW. Prolonged mechanical ventilation not only increases the risk of diaphragm dysfunction, but diaphragm dysfunction may further prolong mechanical ventilation duration and increase the risk of weaning failure. These two factors may interact and jointly promote the development and progression of ICU-AW (49–51).

This study showed that the rates of change of rectus femoris thickness (RF-MLT) on D5 and D7 and the rate of change of end-expiratory diaphragm thickness (DTee) on D7 were significantly different between the two groups, suggesting that dynamic changes in rectus femoris thickness and end-expiratory diaphragm thickness may be more sensitive for identifying ICU-AW.

Only the dynamic changes of RF-MLT and DTee at specific time points showed significant intergroup differences, while RF-CSA, VI-MLT, and DTei did not. This may reflect that these parameters have relatively lower sensitivity to ICU-AW-related muscle atrophy in this elderly population, or that the sample size was insufficient to detect smaller effect sizes for these parameters. Additionally, variability in probe compression pressure during ultrasound measurements may have introduced minor measurement errors, potentially affecting the precision of thickness assessments.

Furthermore, rectus femoris thickness demonstrated better discriminative ability than cross-sectional area, which may be related to its measurement characteristics. Compared with cross-sectional area, thickness measurement is simpler to perform, less affected by factors such as probe compression pressure and imaging plane angle, and thus has better reproducibility and stability, allowing earlier detection of subtle changes in muscle structure. In contrast, cross-sectional area measurement is more susceptible to operator experience and measurement conditions, which may reduce its sensitivity.

Regarding diaphragm parameters, this study showed that the rate of change of end-expiratory diaphragm thickness was more closely associated with ICU-AW, whereas no significant intergroup difference was observed for end-inspiratory diaphragm thickness. The reason may be that the diaphragm is in a relatively resting state during end-expiration, which is less affected by the patient's breathing effort and cooperation, thus providing a more stable and objective reflection of true structural changes in the diaphragm. This suggests that end-expiratory diaphragm thickness may be a superior indicator for assessing diaphragm atrophy and identifying the risk of ICU-AW in critically ill patients. Furthermore, fluid balance, tissue edema, and ventilator conditions may also influence serial muscle and diaphragm thickness measurements; however, these factors were not standardized or adjusted for in the present analysis. Indeed, fluid overload has been shown to artifactually enlarge muscle dimensions on ultrasound, potentially confounding the interpretation of serial muscle thickness measurements (52).

### 4.4 Correlation between muscle changes and muscle strength

ICU-AW not only prolongs mechanical ventilation duration, increases weaning failure rates, and extends ICU stay, but is also closely associated with impaired physical function, reduced long-term quality of life, and increased mortality risk. Previous studies have shown that the incidence of ICU-AW is high in patients requiring mechanical ventilation for more than 4–7 days, and can be even higher in patients with sepsis, septic shock, and multiple organ dysfunction syndrome (51, 53, 54).

The results of our correlation analysis showed that the rates of change of rectus femoris thickness, rectus femoris cross-sectional area, and vastus intermedius thickness on D5 and D7 were all negatively correlated with the MRC score, while the rate of change of end-expiratory diaphragm thickness on D7 showed the strongest correlation with the MRC score (*r* = −0.413, *P* < 0.001). This finding indicates that a greater degree of muscle atrophy is associated with lower muscle strength. Notably, the strongest correlation was observed between diaphragm changes and muscle strength in this cohort, suggesting a potential association between respiratory muscle structural changes and overall muscle strength. However, as this finding is correlational, it does not establish a causal role for the diaphragm in the development or progression of ICU-AW. The underlying mechanism may be related to microcirculatory dysfunction and metabolic disorders under critical illness conditions (55). Activation of the inflammatory response, vasodilation, and increased vascular permeability can lead to tissue edema, local cytokine release, and impaired blood perfusion and oxygen delivery (56, 57). Meanwhile, compression of muscles and nerves due to tissue edema, as well as neuronal injury, axonal degeneration, and chronic depolarization of terminal motor nerve axonal membranes caused by hypoperfusion and ischemia-hypoxia, may all contribute to the development of muscle weakness (58). Based on these findings, we suggest that diaphragmatic ultrasound may provide useful information for assessing both respiratory muscle structure and systemic muscle functional status in critically ill patients.

### 4.5 ROC analysis and clinical significance

The ROC curve and AUC are commonly used to evaluate diagnostic accuracy. An AUC of 0.5–0.7 indicates low, 0.7–0.9 moderate to good, and >0.9 high discriminative efficacy (59). In this study, statistical significance was defined as the 95% CI of the AUC not including 0.5, and no formal pairwise comparison between AUCs was performed. Our ROC results showed that the D7 relative changes in both RF-MLT and DTee demonstrated statistically significant discriminatory ability for ICU-AW, with AUCs of 0.672 and 0.693, respectively. The optimal cutoff values were a decrease ≥3.45% for RF-MLT (sensitivity: 50.0%, specificity: 86.4%) and a decrease ≥6.07% for DTee (sensitivity: 73.1%, specificity: 59.1%). The D7 relative change in DTee showed the highest numerical AUC among the evaluated parameters. As no formal pairwise comparison between AUCs was performed, these findings should be interpreted descriptively. The combined use of DTee and RF-MLT warrants further investigation. but the difference from RF-MLT was small and not formally tested. Given the AUC of 0.693 and specificity of 59.1%, its discriminatory and clinical utility should be interpreted as limited. The combined use of DTee and RF-MLT warrants further investigation in larger cohorts with formal AUC comparison.

## 5 Conclusion

Dynamic monitoring of quadriceps and diaphragm thickness using bedside ultrasound can effectively reflect the progressive atrophy of peripheral skeletal muscle and respiratory muscle in elderly critically ill patients during ICU stay. Compared with baseline muscle parameters, the rates of change of RF-MLT and DTee are more closely associated with the occurrence of ICU-AW and show good correlation with MRC scores. ROC analysis showed that the D7 relative change in DTee had the numerically highest AUC among the evaluated parameters, with an optimal cutoff value of a decrease ≥6.07% (sensitivity 73.1%, specificity 59.1%). However, as no formal pairwise comparison between AUCs was performed, these findings should be interpreted descriptively and warrant further validation in larger prospective studies. Given its non-invasive, real-time, and reproducible nature, bedside ultrasound is suitable for early screening and dynamic assessment of muscle and diaphragm changes in elderly ICU patients. Combined monitoring of multiple muscle parameters may help improve the early identification rate of ICU-AW and provide an objective basis for clinical decision-making.

## 6 Limitations

This study has several limitations. First, this was a single-center study with a relatively limited sample size, which may affect the generalizability of the findings. Second, we only observed short-term changes during ICU stay and did not perform long-term follow-up, so we cannot evaluate the relationship between muscle changes and long-term functional outcomes. Third, the ultrasound examiners were not blinded to patients' clinical status, and all measurements were performed by the same two trained operators, which may have introduced measurement and operator-dependent bias. Fourth, MRC assessments at Day 7 required patients to be alive, still in the ICU, and able to cooperate, which may have introduced selection and survivor bias. Fifth, we did not measure diaphragmatic excursion or calculate the thickening fraction; therefore, diaphragmatic thickness changes should be interpreted as structural alterations rather than functional impairment. Sixth, ventilator settings were not recorded, and we did not adjust for fluid balance, tissue edema, or ventilator conditions, all of which may influence serial muscle and diaphragm thickness measurements. Seventh, no formal pairwise comparison between AUCs was performed, and the ROC cutoffs were derived and evaluated in the same cohort without internal or external validation; therefore, the predictive findings should be interpreted descriptively and require validation in an independent population. Furthermore, the AUC values were generally at a moderate-to-low level, suggesting that further optimization of the prediction model is needed. Future multi-center studies with larger sample sizes, blinded assessments, and multiple operators are warranted to validate our findings.

## Data Availability

The original contributions presented in the study are included in the article/Supplementary Material, further inquiries can be directed to the corresponding author/s.
